# Early osseointegration of micro-arc oxidation coated titanium alloy implants containing Ag: a histomorphometric study

**DOI:** 10.1186/s12903-022-02673-6

**Published:** 2022-12-22

**Authors:** Mingchao Ding, Jin Shi, Weiqi Wang, Dechao Li, Lei Tian

**Affiliations:** 1 State Key Laboratory of Military Stomatology and National Clinical Research Center for Oral Diseases and Shaanxi Clinical Research Center for Oral Diseases, Department of Oral and Maxillofacial Surgery, School of Stomatology, The Fourth Military Medical University, No. 145 Changle Xi Road, Xi’an, 710032 People’s Republic of China; 2 Qingdao Stomatological Hospital Affiliated to Qingdao University, No. 17 Dexian Road, Shinan District, Qingdao, 266001 Shandong Province People’s Republic of China

**Keywords:** Micro-arc oxidation, Ag, Titanium alloy implants, Osseointegration

## Abstract

**Background:**

This study aimed to evaluate bone response to micro-arc oxidation coated titanium alloy implants containing Ag.

**Methods:**

144 titanium alloy implants were prepared by machine grinding and divided into three treatment groups as following, SLA group: sand-blasting and acid-etched coating; MAO group: micro-arc oxidation without Ag coating; MAO + Ag group: micro-arc oxidation containing Ag coating. Surface characterization of three kind of implants were observed by X-ray diffraction, energy dispersive X-ray spectrometer, scanning electron microscopy, High Resolution Transmission Electron Microscope and roughness analysis. The implants were inserted into dog femurs. 4, 8 and 12 weeks after operation, the bone response to the implant to the bone was evaluated by push-out experiment, histological and fluorescent labeling analysis.

**Results:**

MAO + Ag group consisted of a mixture of anatase and rutile. Ag was found in the form of Ag_2_O on the surface. The surface morphology of MAO + Ag group seemed more like a circular crater with upheaved edges and holes than the other two groups. The surface roughness of MAO and MAO + Ag groups were higher than SLA group, but no statistical difference between MAO and MAO + Ag groups. The contact angles in MAO + Ag group was smallest and the surface free energy was the highest among three groups. The maximum push-out strength of MAO and MAO + Ag groups were higher than SLA group at all time point, the value of MAO + Ag group was higher than MAO group at 4 and 8 weeks. Scanning electron microscopy examination for the surface and cross-section of the bone segments and fluorescent labeling analysis showed that the ability of bone formation and osseointegration in MAO + Ag group was higher than that of the other two groups.

**Conclusion:**

The micro-arc oxidation combination with Ag coating is an excellent surface modification technique to posse porous surface structure and hydrophilicity on the titanium alloy implants surface and exhibits desirable ability of osseointegration.

## Background

Titanium and its alloys have been widely used in the stomatology field because of their better biocompatibility, mechanical properties, and corrosion resistance to other metallic biomaterials [1], i.e., an intimate and direct contact with bone by a cement-free connection at the light-microscopic level. A consensus report showed moderately rough and rough surfaces provided enhanced bone integration compared with smooth and minimally rough surfaces [2] and a recent study comparing surfaces with defined.

microroughness confirmed beneficial effects of moderately rough surfaces on osteoblast differentiation and migration [3]. Human and animal histomorphometric evalutions have shown greater bone-to-implant contact at acid-etched implants [4]. However, in some cases, the titanium dental implants still have poor osteointegration [2, 5]. To enhance the mechanical and biological properties of the implants, surface modifications have been widely studied with the objective to increase bone-to implant contact (BIC), particularly in low-density bone tissue areas.

A series of surface modifications has been developed and applied on marketed implants by different subtracting and additive methods, including grit-blasting, plasma spraying, acid etching by mineral acids, micro-arc oxidation (MAO), calcium-phosphate coatings or several combinations of these techniques, e.g., combined grit-blasted/acid etched surfaces [6–12]. These methods have produced excellent clinical results, acquiring early osseointegration of implants, immediate load on implants and implantation under poor bone conditions [13, 14].

MAO is an electrochemical surface modification technique using high voltages (several hundred volts) to fabricate porous and thick oxide coatings on metals and to incorporate calcium (Ca) and phosphorus (P) ions into the surface layer (Ca–P coatings) [15]. This layer can serve as a transition layer to enhance the adhesion strength of post-prepared coatings, because the porous topography formed by MAO largely increase the contact area between coatings and substrates [16, 17]. Furthermore, it can act as a physical protective layer to ensure the crystallization process of calcium phosphates during coating preparation [18]. The MAO layer also changes several surface properties of the implants such as crystal structure, chemical composition and roughness, which can improve the stability of implants. Ag and its compounds have been incorporated into the surface of medical devices because of antimicrobial activity. Most of the studies have focused on the molecular mechanisms underlying the osseointegration of MAO-treated implants, but appropriate in vivo models investigating the osseointegration ability associated with bone formation and resorption around MAO + Ag-treated implants have been less demonstrated.

The aim of our experiment was to study the osseointegration ability of titanium implants which incorporated Ag into the Ca–P coating using MAO method in vivo. The null hypothesis of the study is that there are not any significant differences among the groups.

## Materials and methods

### Implants materials

144 titanium alloy implants (TLM) samples (Ti–3Zr–2Sn–3Mo–25Nb, Northwest Institute for Nonferrous Metal Research, China) with a length of 11.0 mm and a diameter of 3.3 mm were used in our study. All implants were cleaned by ultrasonic rinsing for 5 min, then in distilled water for an additional 5 min to degrease and remove contaminants from the surface. These samples were sandblasted with large 0.3–0.4 mm Al_2_O_3_ (0.8 KPa) grit and ultrasonically cleaned in consecutive washes of acetone, ethyl alcohol, and distilled water. The samples were then divided into three groups with 48 samples in each. SLA group were acid-etched with HCl/H_2_SO_4_ at 60 °C for 90 min. MAO group were anodized in an electrolytic solution containing 15 g/L ammonium phosphate dibasic (NH_4_H_2_PO_4_), 2 g/L potassium hydroxide and 20 g/L calcium acetate monohydrate ((CH_3_COO)_2_CaH_2_O) by MAO treatment (voltage, 465 V; pulse frequency, 600 Hz; oxidizing time, 6 min) and formed of Ca–P coatings without Ag. MAO + Ag group were Ca–P coatings formed MAO containing Ag that was introduced in the form of AgNO_3_ at concentrations of 0.004 mol/L.

### Surface characterization

The phase compositions of the implants were analyzed by X-ray diffraction (XRD, Empyrean, PANalytical B.V., Netherlands). Scanning Electron Microscopy (SEM, Supra 55, Zeiss, Germany) was used to observe the implant surface morphology. The elemental concentrations of the implant surfaces were quantified by Energy Dispersive X-ray Spectrometer (EDS). High Resolution Transmission Electron Microscope (HRTEM, TECNAI-F30, Philips-FEI, Holland) was used to identify in the form of Ag on the surface of MAO + Ag group.

The surface parameters measured by the step profiler (probe-type surface profiler) (DektakXT, Germany) were used to quantify the roughness of implants, including the roughness average or the mean height of peaks (R_a_), the 3-dimensional root-mean-squared roughness (R_q_) and the maximum height of the profile from highest to lowest point (R_t_). Lastly, the contact angles and surface energies were measured by the contact angle meter (DSA100, Germany). Briefly, we chose deionized water and methylene iodide as the detection liquid. 10 µL of droplets was prepared onto the sample surface and measured five times at different locations of each sample [19]. The surface free energy (SFE) was calculated by using Owens-Wendt method [20].

### Animals

The animal experiment was approved by the Ethics Committee of Jiamusi University (2018-132). This study conformed to the Arrived guidelines. 24 mongrel male dogs with an age of 2–4 years and weighing 30 ± 5 kg were purchased from Jiamusi University Animal Laboratory. 48 of each kind of implants were placed in the dog’s left and right femurs and evaluated after 4, 8 and 12 weeks following the implantation surgery.

### Surgical procedures

All animals were operated under general anesthesia during the surgery. Anesthesia was applied using ketamine (Hypnorm VetR, Janssen, Saunderton, England) at a dose of 2–4 mg/kg body weight. The surgical site of animals was shaved and sterilized by iodine. The surgical site was incised with a scalpel. The medial aspect of the proximal metaphysis was exposed since skin, muscles and periosteal layers were separately pulled away from the surgical site. Three implant cavities at intervals of 3 mm were prepared in each femoral metaphysis using 2.0 mm pilot drill at 1200 rpm and then prepared by an expanding drill (φ2.8 and 3.3 mm) at 800 rpm. Three kinds of implants were installed into the left femur and operated the same process on the right leg. The surgical wound was closed routinely with 4 - 0 nylon sutures. Sutures of skin were removed from wounds in 10 days after the implantation surgery. The animals were injected with diclofenac sodium and cefotaxime sodium for 5 days to control postoperative pain and prevent postsurgical infection [21]. The animals at 4, 8 and 12 weeks were randomly divided into two groups (push-out experiment and fluorochrome labeling experiment).

### Push-out experiment

The femora were harvested with special care not to impair the bone surfaces and embedded in a custom-made mold using an autopolymer resin at each time point. The blocks were sprayed with saline solution every 15 min to prevent them from drying and incised to expose both ends of the implants to expose a flat-bottomed surface that was parallel to the implant platform. Prior to the push-out test, the direction of each implant was measured against two axes under an incident microscope (Acoustic Microscope, Olympus Optical, Tokyo, Japan). All specimens were tested in a universal testing machine (CSS-44,500, China) to obtain the push-out values. The testing machine was equipped with a 2000 N load cell that contained a 2.5 mm diameter custom-made stainless-steel pushing rod. The axial load on the implant was applied at a cross-head speed of 1 mm/min. During constant pushing out, displacement of the implant and the load were simultaneously recorded at a sampling rate of 4 Hz. The load-displacement curve was recorded using x-t recording software. The push-out test value was determined as the breakpoint load.

### Fluorochrome labeling

For fluorochrome sequential labeling, three fluorescent bone markers were administered and incorporated into newly formed bone. Tetracycline hydrochloride (10 mg/ml, 30 mg/kg) was subcutaneously injected into the dogs at 3 weeks, alizarin red (10 mg/ml, 30 mg/kg) at 1 weeks and calcein (10 mg/ml, 5 mg/kg) at 3 days before the sacrifice, respectively [22].

### Histological preparation

Each specimen containing three implants was trimmed into three blocks by a microtome (LeicaSP 1600, Milan, Germany). All the blocks of dog femurs were prepared for undecalcified specimens. Each block with one implant was dehydrated in a graded series of ethanol from 75 to 100%, and then infiltrated with polymethyl methacrylate (PMMA) and finally cut along the long axis of the implant into 100-µm-thick bone-implant Sect.  [23].

### Confocal laser scanning microscope (CLSM) observation

Fluorescently stained bone-implant sections were observed by confocal laser scanning microscope (Olympus FluoViewer1000, Japan) to analyze the osteogenesis in bone-implant interface. Excitation wavelength of tetracycline hydrochloride was 365–436 nm and emission wavelength was 570 nm. Excitation wavelength of alizarin red was approximately 530–580 nm, and emission wave length was 600–645 nm. Excitation wavelength of calcein was approximately 495 nm, and emission wave length was 515 nm [24]. The image was processed via a matched computer image software viewer (Olympus FluoViewer1000, Japan).

### Statistical analysis

Statistical analysis was performed using SPSS19.0 software (SPSS, Chicago, USA). All data were normally distributed and expressed as mean ± standard deviation (s.d.). The results were compared using the One-Way ANOVA followed by Dunnett multiple comparison test among the groups at each time point. *p*-value < 0.05 was considered significant.

## Result

### Surface analysis

**Fig. 1:**
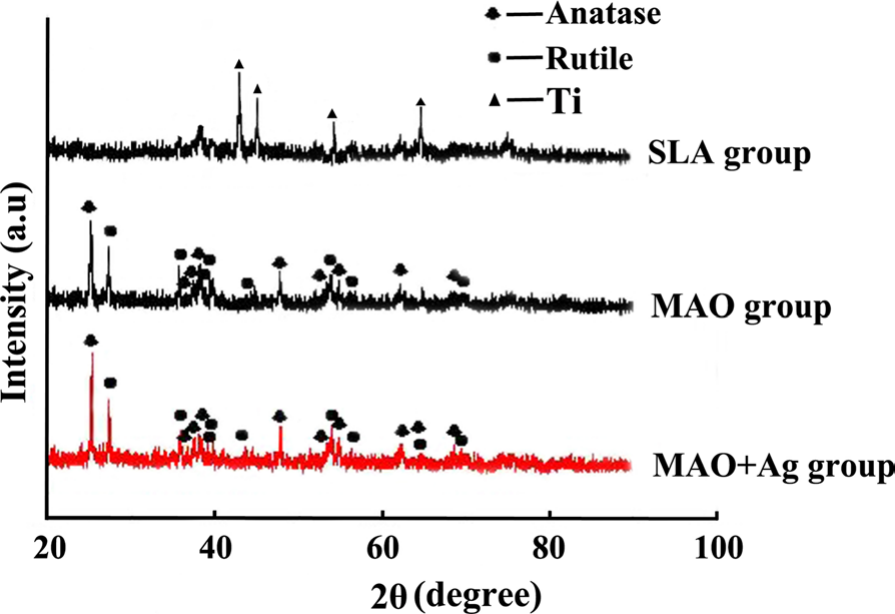
XRD patterns of three groups coatings

The XRD patterns showed that MAO and MAO + Ag groups coating mainly consisted of a mixture of anatase and rutile (the natural form of TiO_2_). The SLA group coating was composed of Ti (Fig. 1).

**Fig. 2:**
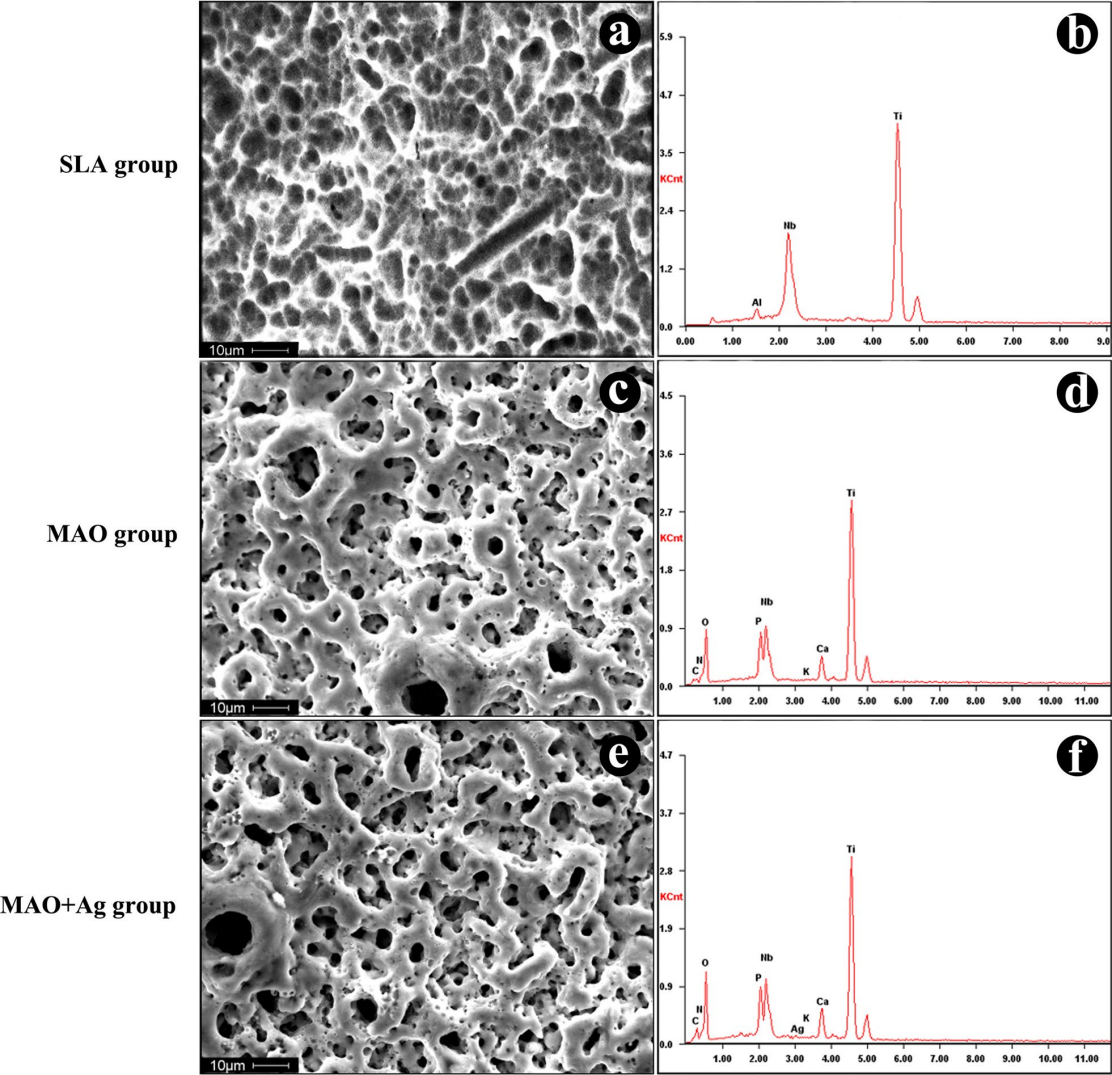
SEM images of surface morphology for three groups (**a,** **c,** **e**). EDS spectrum analysis of three groups coatings (**b,** **d,** **f**)

**Fig. 3:**
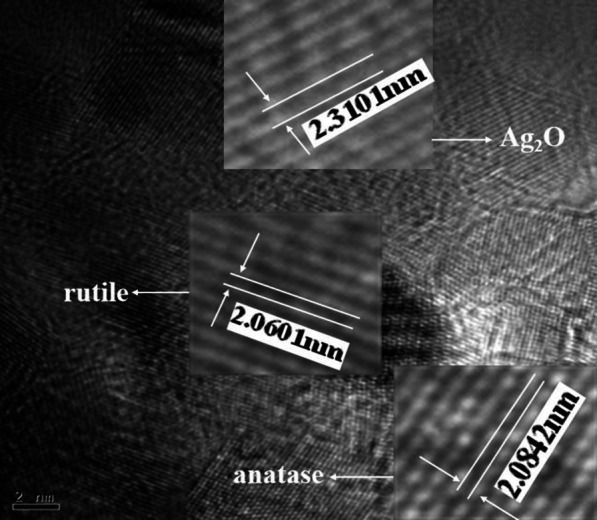
HRTEM morphology of MAO + Ag group surface

All kinds of implants had porous surface structures, but the surface morphology differed drastically among the groups. Both MAO and MAO + Ag groups had porous surface structures with the pore diameter ranging from a submicron scale on the surface, while SLA group exhibited relatively smoother surfaces. Furthermore, in the MAO + Ag group, the surface morphology seemed more like a circular crater with upheaved edges and holes connected with each other (Fig. 2a, c, e). EDS results revealed that Ca and P were incorporated into the surface layer after modification with MAO technique. A wave crest of Ag was observed in MAO + Ag group (Fig. 2b, d, f). Meanwhile, HRTEM showed that Ag was found in the form of Ag_2_O on the surface of MAO + Ag group (Fig. 3).

Regarding the surface roughness analysis, R_a_, R_q_ and R_t_ in MAO and MAO + Ag implants were significantly higher than SLA group (*p* < 0.05), but there were no statistical difference between MAO and MAO + Ag implants (*p* > 0.05). The contact angles in MAO + Ag group was smallest among three groups (*p* < 0.05), and that in MAO group was lower than SLA groups. The SFE in MAO + Ag group was the highest among the groups (*p* < 0.05), and that in MAO group was higher than SLA groups (*p* < 0.05) (Table 1).

**Table 1:** Surface roughness parameters, contact angles and SFE of three groups

Sample	R_a_ (µm)	R_q_ (µm)	R_t_ (µm)	Contact angle (°)		SFE (m JM-2)
				Water	Methylene	
SLA group	0.50 ± 0.09	1.12 ± 0.15	3.27 ± 0.66	100.4 ± 2.21	78.08 ± 5.79	27.65
MAO group	0.90 ± 0.07*	2.14 ± 0.20*	5.81 ± 0.83*	10.3 ± 1.53*	24.57 ± 4.59*	52.40*
MAO + Ag group	0.91 ± 0.17*	2.15 ± 0.43*	6.15 ± 0.97*	5.8 ± 0.92*^#^	16.92 ± 4.27*^#^	78.39*^#^

*Means the difference between the group and SLA group

^#^Means the difference between the group and MAO group

### Maximum push-out force

**Fig. 4:**
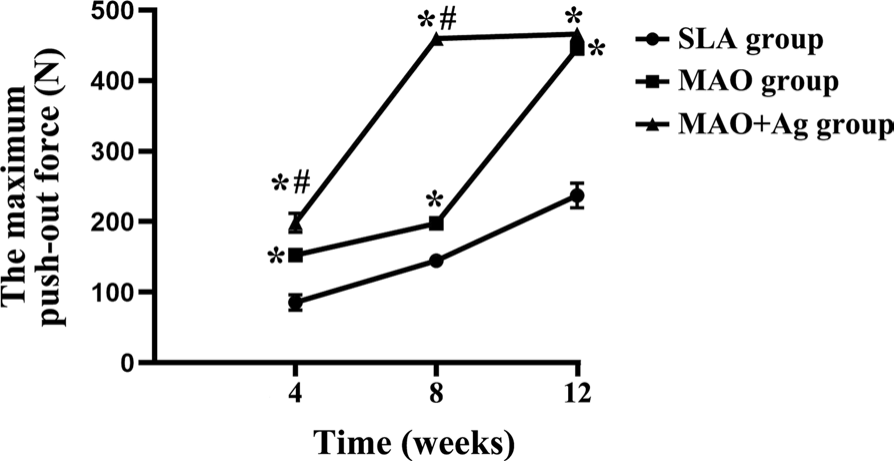
The mean peak values for push-out strength of three groups. *Means the difference between the group and SLA group; # means the difference between the group and MAO group

The maximum strength of three groups were 85.53 ± 10.74 N, 144.66 ± 7.20 N and 237.27 ± 17.41 N respectively at 4 weeks after implantation. The maximum strength of three implant groups were 152.56 ± 6.39 N, 197.53 ± 3.17 N and 445.83 ± 3.35 N respectively at 8 weeks after implantation. The maximum strength of three implant groups were 198.56 ± 13.51 N, 459.65 ± 2.72 N and 466.01 ± 4.11 N respectively at 12 weeks after implantation. Statistics showed the maximum push-out strength of MAO and MAO + Ag groups were higher than SLA group at all time point (*p* < 0.05). The value of MAO + Ag group was higher than MAO group at 4 and 8 weeks (*p* < 0.05) (Fig. 4).

### SEM for the surface and cross-section morphology of the bone segments after the push-out test

**Fig. 5:**
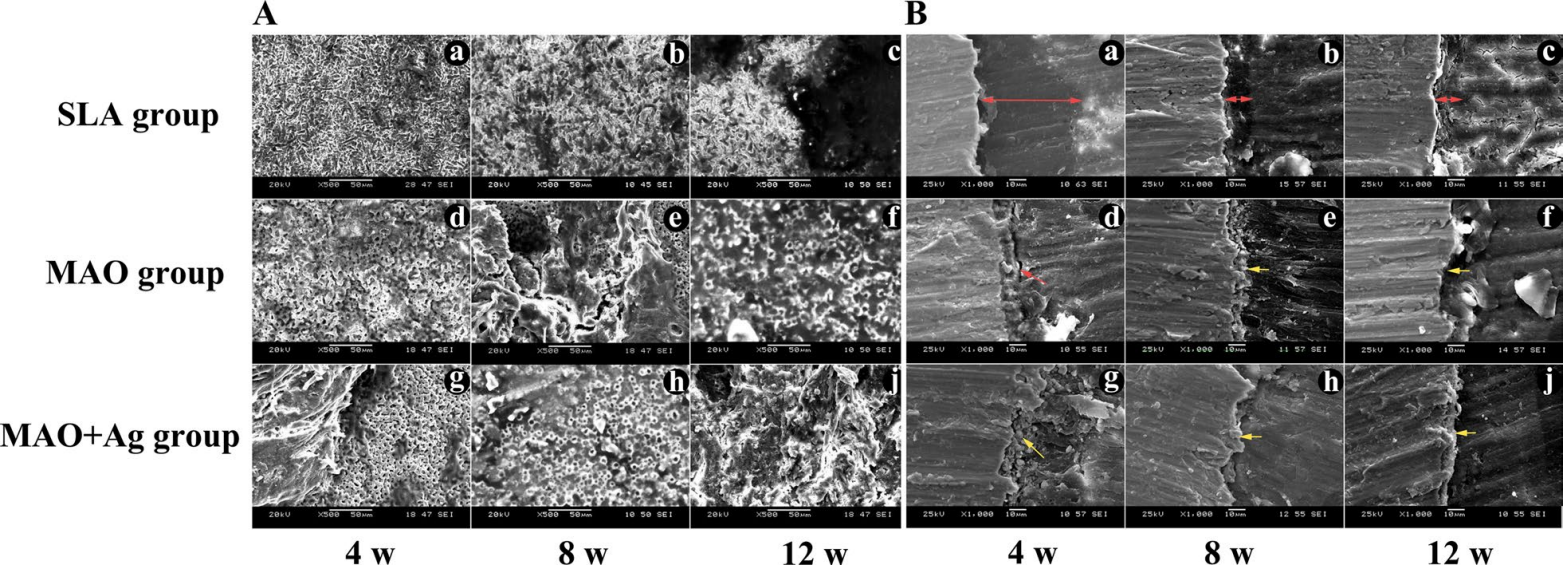
SEM images of the surface (**A**) and cross-section (**B**) morphology of the bone segments after the push-out test at 4, 8 and 12 weeks. Red arrow means the gap between implant and bone; Yellow arrow means the contact area between the implant and the bone

The surface of the three groups after push-out experiment were analyzed by SEM (Fig. 5A). At 4 weeks, there was very little bone component on the surface of SLA group (Fig. 5A(a)), while the bone tissue began to grow toward and spread over the porous structure in MAO and MAO + Ag group (Fig. 5A(d, g)). At 8 weeks, bone tissue was still not obvious in the SLA group (Fig. 5A(b)), the pores on the surface of MAO group were still visible and not fully filled with bone tissue (Fig. 5A(e)), while the pores on the surface of MAO + Ag group were already fully filled with bone (Fig. 5A(h)). At 12 weeks, we could see that the bone remained on the surface of SLA group (Fig. 5A(c)). Most of pores were filled with bone on the surface of MAO implants (Fig. 5A(f)). Meanwhile, in MAO + Ag group, the bone tissue was filled in the pores throughout the hole implant samples (Fig. 5A(j)).

The cross-section of the bone segments were analyzed by SEM (Fig. 5B). Although the gap between implant and the surrounding bone became narrow over time in all three groups, we found there was still a slit here after 12 weeks of healing in the SLA group (Fig. 5B(c)). A slit was also observed in MAO group at 4 weeks after healing (Fig. 5B(d)), but it was less clearly observed at 8, 12 weeks (Fig. 5B(e)). In the MAO + Ag group, the coating was particle-like and presented more compacted than the other two groups at 4, 8 and 12 weeks (Fig. 5B(g, h, j)).

### CLMS analysis

**Fig. 6:**
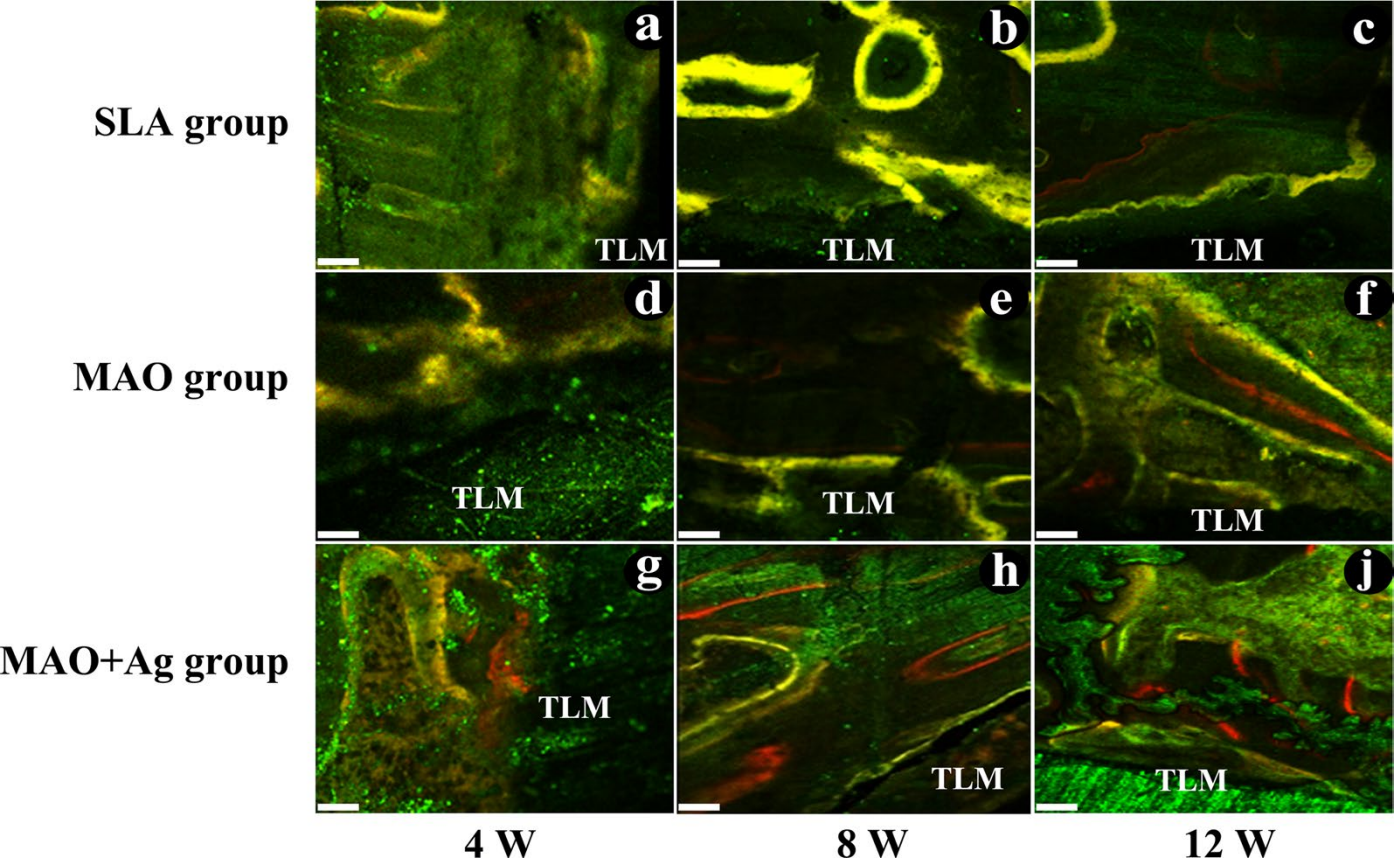
CLSM images of three groups at 4, 8 and 12 weeks. Bars = 100 μm

All three groups showed stronger fluorescence bands over time. At 4 weeks, SLA group showed no obvious fluorescence bands around the implants, intermittent yellow and green bands were observed around the implants in MAO group, while MAO + Ag group manifested a continuous fluorescence band which was contacted closely with the implant (Fig. 6a, d, g). At 8 weeks, SLA and MAO group showed a relatively narrow and irregular yellow and green fluorescent band (Fig. 6b, e). MAO + Ag group manifested yellow-green and red band. The most obvious phenomenons were that the green bands developed in two directions: towards and away from the implant and a portion of the fluorescent areas began to move away from implant, indicating that osteogenesis was basically completed in these areas (Fig. 6h). At 12 weeks, MAO + Ag group showed much more red bands than the other two groups and the yellow fluorescent band at the contact area had almost disappeared (Fig. 6c, f, j).

## Discussion

The biomedical titanium alloy (TLM) was recently developed using non-toxic alloying elements to achieve a low elastic modulus, good strength, and good processability, which makes it an ideal candidate for hard tissue replacement [25]. To achieve better bioactivity, bioactive ceramic coatings have been used on orthopedic and dental implants, which offer the possibility of combining the strength of the metals with the bioactivity of the ceramics [26].

Bone growth on titanium materials surface mainly depends on the surface microstructures [27]. Many surface modifying techniques have been improved [28, 29]. MAO coatings of implants is one of the most effective ways to improve the surface structure of endosteal implants because of the good adhesion to the substrates and against the release of metal ions from substrates as a chemical barrier [30–33]. The surface of MAO implants became roughness, which increased the contact area with bone and influenced bacterial adhesion. A problem for titanium implant is lack of antibacterial property which make them fragile to bacterial infection [34]. Bacterial infection is a severe complication for titanium implants and it may occur immediately after implantation surgery as a result of bacteria containment or after long-term use as a result of bacteria biofilm colonization on dental implants, which will subsequently lead to periimplantitis, or even implant failure [35]. the treatment modalities include mechanical debridement [36], the administration of chlorhexidine [37], air polishing with glycine or bicarbonate powder [38] and ozonized water [39]. Meanwhile, various inorganic antiseptics, including Cu, Zn, Mn and Ag have been introduced into titanium coatings to strengthen their antibacterial activity [40, 41]. Ag reacts with both microbial DNA and the sulfhydryl groups of the metabolic enzymes of the bacterial electron transport chain, which induces the inactivation of bacterial proteins. It has been reported the coatings obtained in an Ag-containing solution showed an in vitro antibacterial activity [42]. Zhang et al. [27] showed that incorporation of Sr and Ag could not affect coating micromorphology and the effects of Sr and Ag on coating biological activities might only attribute to their own or synergistic activities.

The null hypotheses of this study were rejected. In our experiment, MAO was used to provide a good combination of porous oxide layers on TLM, and then, Ag was successfully incorporated over the porous coating via AgNO_3_ at concentrations of 0.004 mol/L. We analyzed the surface elemental concentrations in vitro, SLA group coating was composed of Ti, and a similar mixture of anatase and rutile were observed on MAO and MAO + Ag group surface. We observed the surface of MAO and MAO + Ag implants contained Ca and P though EDS experiment. It may increase the ability of osteogenic capability, because Ca- and P-containing oxide films are more beneficial for initial cell attachment and proliferation, and they can induce higher osteoconduction [43]. Meanwhile, Ag was observed on the surface of MAO + Ag implants in the form of Ag_2_O. SEM results showed the surface physical characteristics of three groups, though a considerable number of pores were observed on MAO and MAO + Ag group surface, which was similar to the surface appearance reported in previous study [44]. Furthermore, the surface morphology of MAO + Ag implants seemed more like a circular crater with upheaved edges and holes connected with each other. The porous surfaces may enhance early implant fixation because bone can grow inside the pores and promote early stages of osseointegration.

The results showed the surface roughness (Ra, Rq and Rt) in MAO and MAO + Ag groups were significantly higher than SLA group, but there were no statistical difference between the MAO and MAO + Ag groups, while the contact angles in MAO + Ag group was smallest among three groups. Contact angle is related to hydrophilicity and the small contact angle indicates high SFE. Higher SFE enhances the cell adhesion in the early stage of cell response and may work by influencing the expression of adhesion-associated molecules [45–47]. Accordingly, the SFE of MAO + Ag group was highest among three groups. The results implied that Ag might increase the hydrophilicity and osseointegration of implants through reducing the contact angle and increasing SFE, although the surface roughness of the MAO and MAO + Ag groups was similar.

Push-out test is a frequently applied method for characterization of contact phenomena. Our study showed the maximum push-out force of MAO and MAO + Ag groups were higher than SLA group at all time points. The value of MAO + Ag group was higher than MAO group at 4 and 8 weeks, but no difference at 12 weeks. The results might probably imply that Ag showed a significant superiority in the early healing of the implants. SEM results showed that the bone tissue began to grow toward and spread over the porous structure in MAO and MAO + Ag groups at 4 weeks, the pores on the surface of MAO + Ag group were already fully filled with bone at 8 weeks, and the bone filled in the pores throughout the hole implant samples at 12 weeks. It was in accordance with the cross-section of the bone segments. MAO + Ag implants presented more compacted osseointegration than the other two groups at 4, 8 and 12 weeks. CLSM results showed MAO + Ag group manifested a continuous fluorescence band which was contacted much closer with the implant than the other two groups at 4 weeks. Moreover, MAO + Ag group showed much more red bands than the other two group and the yellow fluorescent band at the contact area had almost disappeared. It indicated that osteointegration had nearly completed. The results might possibly be attributed to Ag with Ca-P coating porous mutlipore topography providing a better material environment for cell bonding and survival, and could be favourable for Ca phosphate formation. Furthermore, studies showed that the treatment of murine infected burns with silver nanoparticles could increase the rate of healing, decreased scarring and reduced inflammatory cytokines IL-6 expression [48]. Although water is used to reduce the temperature during implant surgery, drilling a hole in the bone could damage the tissue and induce an immune reaction. Therefore, the anti-inflammatory effect of Ag may play an important role during the healing period post implantation. However, it is an animal study, how the micro-arc oxidation containing Ag coating implant performs as compared with other implant in more complex human environments need for further study.

## Conclusion

In summary, the present results provide evidence that Ag plays role in implant healing. The micro-arc oxidation combination with Ag is an expected surface modification technique to posse porous surface structure and hydrophilicity on the TLM surface. It exhibits ability of osseointegration.

## Data Availability

The data and materials collected in this research are available from corresponding author when requested reasonably.
